# The Dark Pituitary: Hemochromatosis as a Lesser-Known Cause of Pituitary Dysfunction

**DOI:** 10.5334/jbsr.2771

**Published:** 2022-06-29

**Authors:** Bert Verberckmoes, Sven Dekeyzer, Karen Decaestecker

**Affiliations:** 1 University Hospital Ghent, BE

**Keywords:** Magnetic Resonance Imaging, Hemochromatosis, Hypogonadotropic hypogonadism, Pituitary, Iron Deposition

## Abstract

**Teaching Point:** Iron deposition in the pituitary gland in patients with primary and secondary hemochromatosis is a lesser-known cause of pituitary dysfunction and results in T2- and T2*-signal loss on MRI, other brain structures, in which iron deposition can be seen, are the choroid plexus and sporadically the circumventricular organs (e.g., the pineal gland).

## Case Presentation

A 42-year-old male with a history of liver cirrhosis and cardiac decompensation, and with evidence of hemochromatosis on liver biopsy and MRI of the liver and heart, was sent for an MRI of the pituitary gland because of hypogonadotropic hypogonadism.

**Figure 1:**
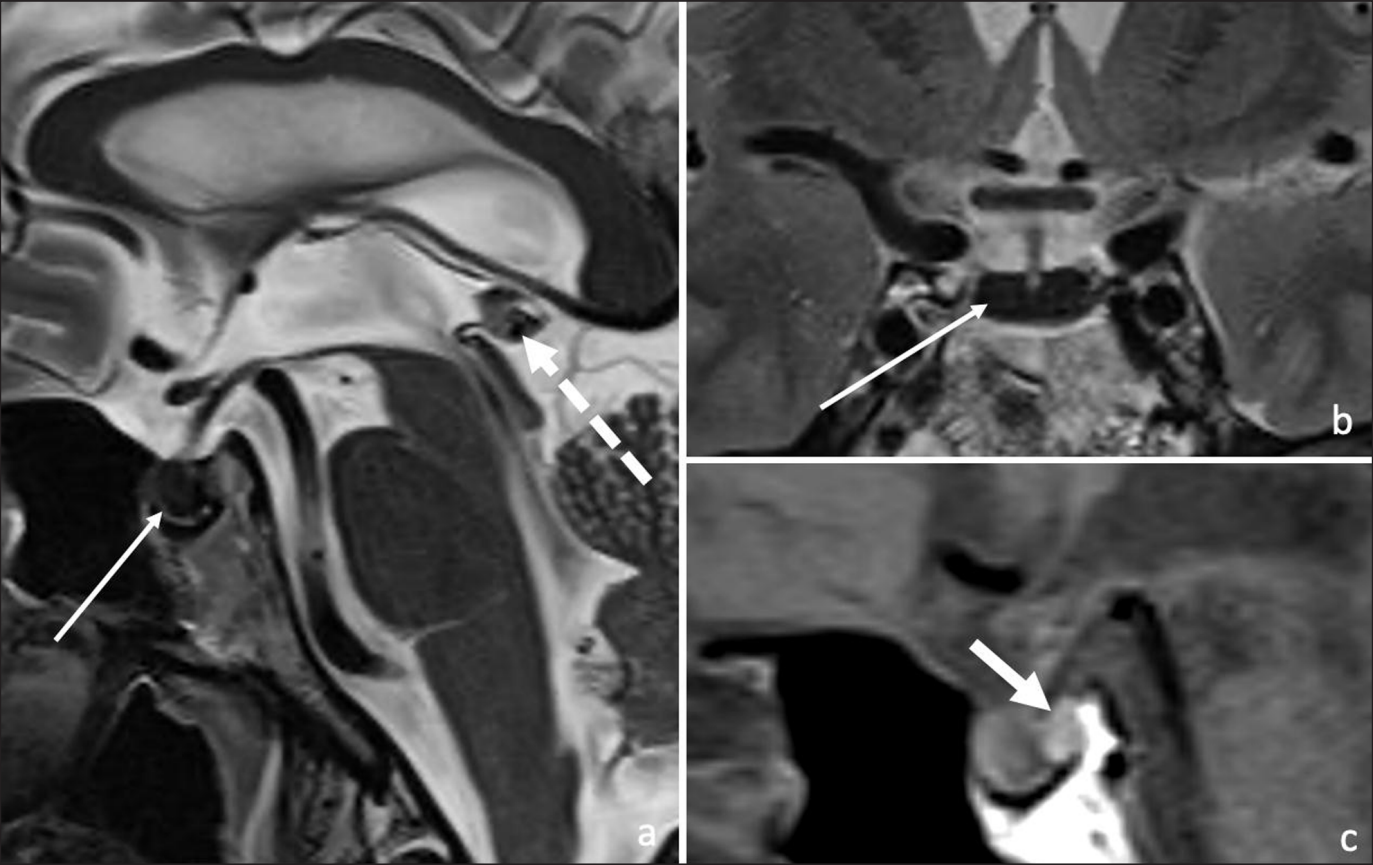


T2-signal loss was seen in the pituitary and the pineal gland with a preserved gland volume and a preserved “neurohypophysis bright spot” (Figure 1, [a] T2 SE, arrow: pituitary gland, dashed arrow: pineal gland, [b] T2 SE, arrow: pituitary gland and [c] T1 SE, arrow: “neurohypophysis bright spot,” note the T1 hypo-intense adenohypophysis). Because of this finding and in light of the patient’s medical history, susceptibility weighed images (SWI) were added to the scan protocol.

**Figure 2:**
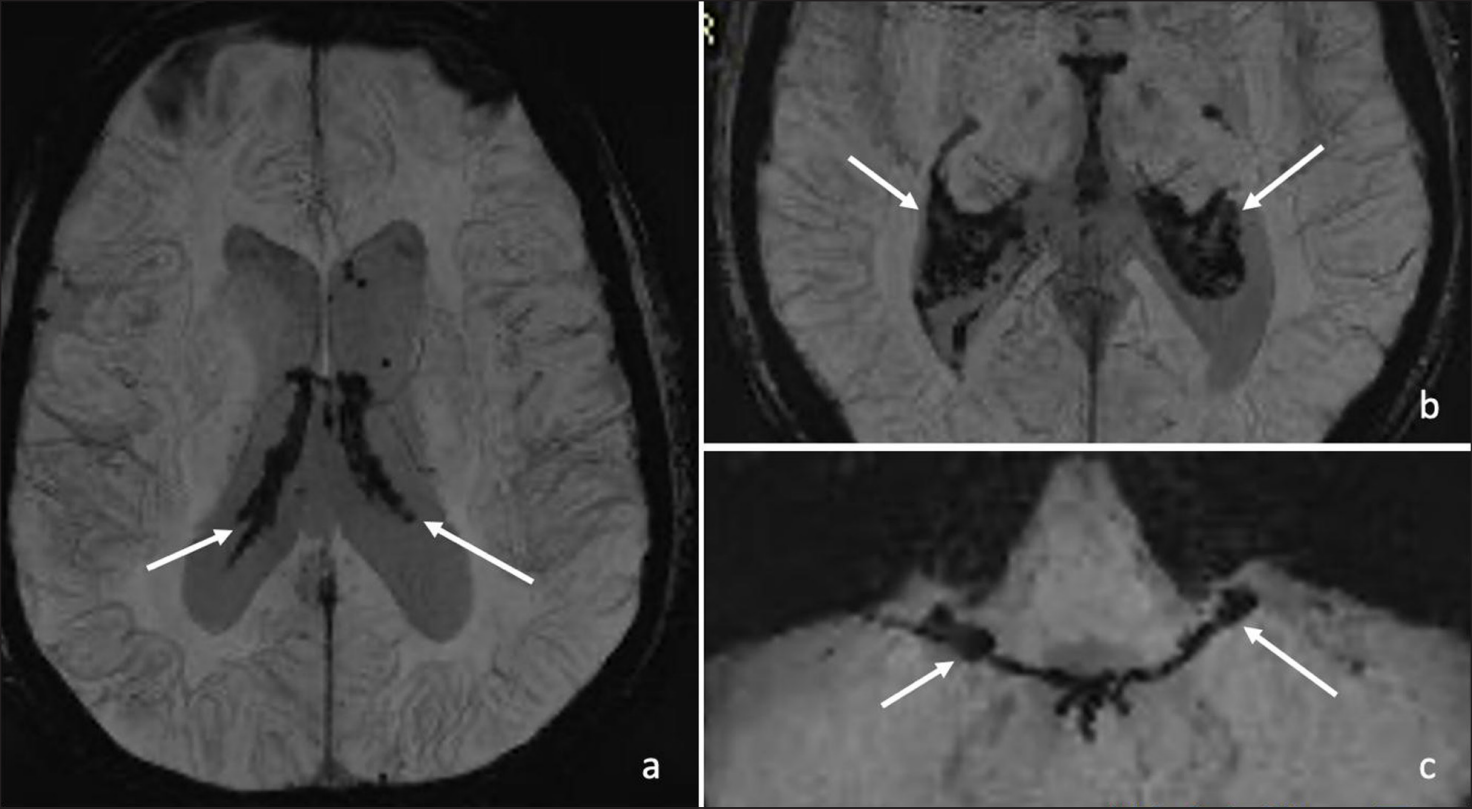


On SWI, pronounced signal loss was present in the choroid plexus, known as the “choroid plexus sign of iron overload” (Figure 2, SWI MIPs [a] arrows: the choroid plexus in the cella media and [b] trigone of the lateral ventricles, and [c] in the fourth ventricle and Lushka foramina).

**Figure 3:**
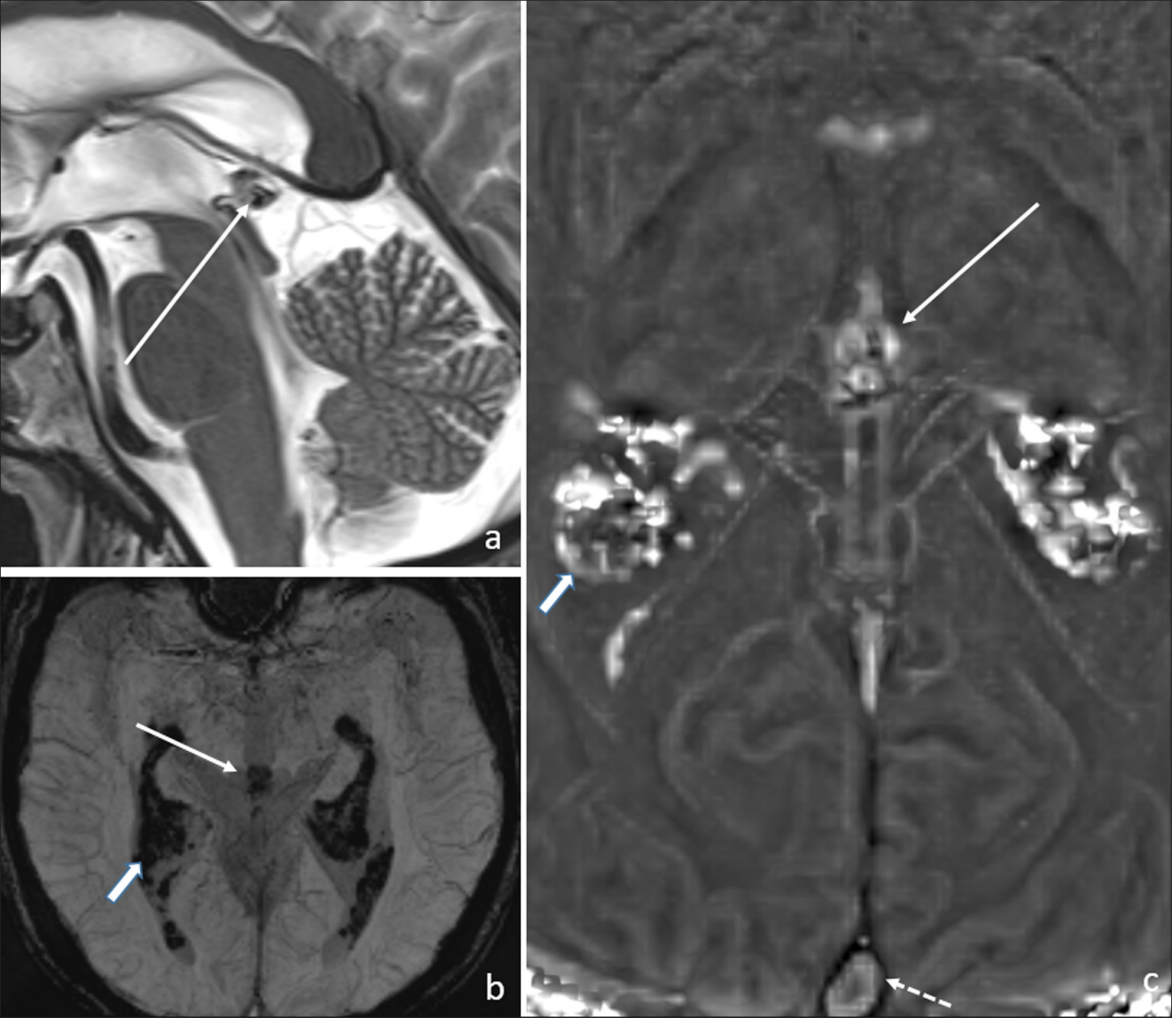


On filtered phase maps the signal loss corresponded to paramagnetic dephasing, indicating the presence of paramagnetic substances such as iron (Figure 3: [a] T2 SE, arrow: pineal gland [b] SWI MIP’s arrow: pineal gland, thick arrow: choroid plexus and [c] filtered phase map, arrow: pineal gland, thick arrows choroid plexus, dashed arrow: sagittal sinus).

## Comment

Hemochromatosis is a systemic disease characterized by increased iron deposition in the body. A distinction is made between primary and secondary hemochromatosis. Primary (or genetic) hemochromatosis is mostly caused by mutations in the gene coding for the HFE-protein. Secondary (or acquired) hemochromatosis is caused by chronic transfusions or iron suppletion to treat chronic anemia or other conditions with an increased erythrogenic need.

Excess iron is deposited in the parenchymal cells of various organs, especially in the heart, liver, joints, skin, and endocrine glands, and eventually leads to cellular damage and organic dysfunction. The brain cells are normally well shielded from pathologic iron accumulation due to the blood brain barrier (BBB). Nevertheless, some CNS structures lay outside the BBB in which iron deposition can occur, such as the pituitary gland, the choroid plexus, and the circumventricular organs. Iron deposition in these structures causes local field inhomogeneities on MRI resulting in signal loss on T2*-images.

In the anterior pituitary gonadotrophic cells seem to be most susceptible to iron deposition, which explains the association between hemochromatosis and hypogonadotropic hypogonadism [1].
